# Designing Chinese hospital emergency departments to leverage artificial intelligence—a systematic literature review on the challenges and opportunities

**DOI:** 10.3389/fmedt.2024.1307625

**Published:** 2024-03-21

**Authors:** Sijie Tan, Grant Mills

**Affiliations:** Bartlett School of Sustainable Construction, Bartlett Faculty of the Built Environment, University College London, London, United Kingdom

**Keywords:** AI, design, Emergency Department, literature review, sensors

## Abstract

Artificial intelligence (AI) has witnessed rapid advances in the healthcare domain in recent years, especially in the emergency field, where AI is likely to radically reshape medical service delivery. Although AI has substantial potential to enhance diagnostic accuracy and operational efficiency in hospitals, research on its applications in Emergency Department building design remains relatively scarce. Therefore, this study aims to investigate Emergency Department facility design by identifying the challenges and opportunities of using AI. Two systematic literature reviews are combined, one in AI and the other in sensors, to explore their potential application to support decision-making, resource optimisation and patient monitoring. These reviews have then informed a discussion on integrating AI sensors in contemporary Emergency Department designs for use in China to support the evidence base on resuscitation units, emergency operating rooms and Emergency Department Intensive Care Unit (ED-ICU) design. We hope to inform the strategic implementation of AI sensors and how they might transform Emergency Department design to support medical staff and enhance the patient experience.

## 1 Introduction

Existing literature on the application of AI in the design of Emergency Department buildings is inadequate, and little is known about AI strategies in emergency settings (e.g., how AI affects medical staff workflows, behaviour and patient needs). More is needed to identify potential challenges and opportunities associated with using both AI and sensors in Emergency Departments and how these might transform the spatial configuration and design of these departments.

Recent advances in computational capabilities, data analysis and algorithmic models have helped establish the era of artificial intelligence (1). There has been swift progress in adopting and developing AI technologies during the COVID-19 pandemic (2) and its use to augment work to create efficiency and supported human error minimisation has been acknowledged.

According to data from the World Health Organisation in 2021 (3), injuries—including both accidental injuries and ones related to violence—account for the demise of approximately 4.4 million individuals worldwide annually, constituting nearly 8% of total deaths. The situation in China is particularly striking: the number of medical visits due to trauma is 62 million annually, with trauma-related fatalities amounting to 700,000–800,000 individuals a year. Trauma has become the fifth leading cause of death (4). Recurrent public safety incidents, such as traffic accidents, construction mishaps, industrial production incidents and fires, lead to consistent surges in casualties and present formidable challenges to China's emergency medical system. Enhancing diagnostic accuracy and efficiency could help streamline hospital operations, thus improving the patient treatment experience (5). We explore how these advances could be combined to design new Emergency Departments (EDs) in China. While many of the applications may be applicable in other countries, this article is focused only on AI adoption in China, where designs differ from those used in the UK, for example, movement away from emergency operating rooms.

Emergency Department often represents the initial point of contact for many patients. The responsiveness and capability of its medical staff critically influence treatment outcomes and so augmenting this setting with AI and sensors could be valuable. We explore how AI methods may support the optimisation of emergency department design in the future. Two systematised literature reviews identify Emergency Department AI strategies and sensor use to optimise China's Emergency Department design. We recommend how advanced standards could interconnect AI, sensors and ED design. This article is structured first to explore the challenges and opportunities in developing an AI-informed design, then to describe the methodology of combining two systematic literature reviews before presenting the challenges, opportunities, and recommendations for using AI-Sensors in Emergency Department design.

## 2 Artificial intelligence (AI) and AI use in medicine (AIM)

Alan Turing first asked, “Can machines think?” (6), then John McCarthy in 1956 formally introduced AI as an academic field (7). AI is a “system that display intelligent behaviour by analysing their environment and taking action—with some degree of autonomy—to achieve specific goals” (8). Artificial intelligence in medicine (AIM) emerged in the early 1970s (9). To encompass early detection and diagnosis, therapeutic interventions, outcome predictions and prognostic evaluations (10). There are ethical considerations, hardware and software security concerns, and technical issues related to societal acceptance and clinical implementation (11). With the maturing of this field, the practical limitations are now better understood. However, the physical build environment and spatial use implications have had limited attention.

In prevention, AI supports the efficiency of potential disease risk factor identification by analysing patients' medical data, medical history, lifestyle choices and genetic profiles. Hamet and Tremblay (12) employ algorithms to analyse electronic health records to identify those with a family history of hereditary diseases or an enhanced risk of chronic conditions. AI is also able to forecast potential complications. For instance, Wong et al. (13) predict the risk associated with idiopathic hemorrhagic ulcers—their recurrence, early identification, risks (such as ulcer perforation) and then early interventions by physicians to increase patient safety. Technologies such as these are likely to have profound impacts on how healthcare systems and services are configured. So, this must be borne in mind when designing and optimising future ED departments.

Medical diagnostics applications of AI are already widespread. They can process and interpret vast amounts of medical data (including medical imagery, pathology reports and clinical trial outcomes). AI-supported medical diagnostics applications can aid physicians in achieving precise diagnoses. For instance, in symptomatic diagnosis. Such as Ansari et al. (14) who employed feed-forward neural networks (FFNNs) and generalised regression neural networks to diagnose liver disease hepatitis viruses. They achieved a disease diagnostic accuracy rate (91.33% and 92%) in seconds, substantially raising both the efficiency and accuracy of the diagnostic process. The implications of earlier and more accurate diagnostics will profoundly affect the healthcare system. It will increase the speed of disease detection and facilitate the placement of such technologies at, or close to, the patient's point of access. The ED provides, therefore, a perfect context within which to explore the application of AI.

Medical treatment applications of AI occurred as early as 1985. Orthopaedic doctors in Canada pioneered “Arthrobot,” marking the world's first use of an AI robot in surgical operations (15). This laid the foundation for subsequent research and applications, such as in treatment decision-making, where Giordano et al. investigated risk stratification of pre-operative patients and the effective categorisation of the health status and severity of diseases in non-surgical patients (16). These capabilities further enhanced the precision and personalisation levels of medical services. This study attempts to make a first attempt at systematising the exploration of AI uses; however, additional interdisciplinary research will be needed to explore the interpretation of literature from various disciplines and across countries. This work builds on an MSc Healthcare Facilities dissertation at UCL.

## 3 Challenges in emergency care

In the 21st century, the field of emergency medical care is facing numerous challenges, such as nursing staff under immense strain (17), waiting lists and the challenges faced by primary healthcare. An increasing number of patients opt to access the healthcare system first through the Emergency Department. The situation is exacerbated due to workforce shortages, limited nursing resources and issues related to social care. The obstacles have been documented. For example, Bijani et al. identified the multiple factors influencing emergency service personnel (18), including professional competence, the work environment, organisational efficiency and ethical concerns. At the same time, Jiménez-Herrera et al. prioritised emotional factors and their influence on emergency nursing decision-making and the emergence of emergency scenarios (19). The limited space in Emergency Departments, patient flow inefficiencies and outdated infrastructure all lead to a cascade of issues, such as overcrowding, extended patient waiting times and decreased satisfaction. Addressing these concerns, the Royal College of Emergency Medicine sounded an alarm, positing that emergency room crowding is a primary impediment to safe, high-quality emergency care (20). Given the complexity of the ED setting, the combined use of advanced sensors and AI could enable rapid learning for ED design optimisation, although few comprehensive implementation examples exist.

According to the World Health Organisation, China has one of the fastest-growing ageing populations globally (21) leading to a rise in the incidence of chronic diseases and the overall medical burden (22). Both non-communicable and infectious disease threats significantly strain China's emergency healthcare system. Correspondingly, the distribution of medical resources in China is uneven (23). Due to financial and technological constraints, many county-level hospitals still need to be developed in their medical service capabilities and patients often opt for larger, more resource-abundant hospitals for treatment when faced with urgent medical needs. This further exacerbates the congestion problem in major hospitals. Therefore, effective AI utilisation may alleviate the uneven distribution of medical resources. This would provide patients with more efficient and accurate medical services (24).

## 4 The systematic literature review methodology

**Figure 1:**
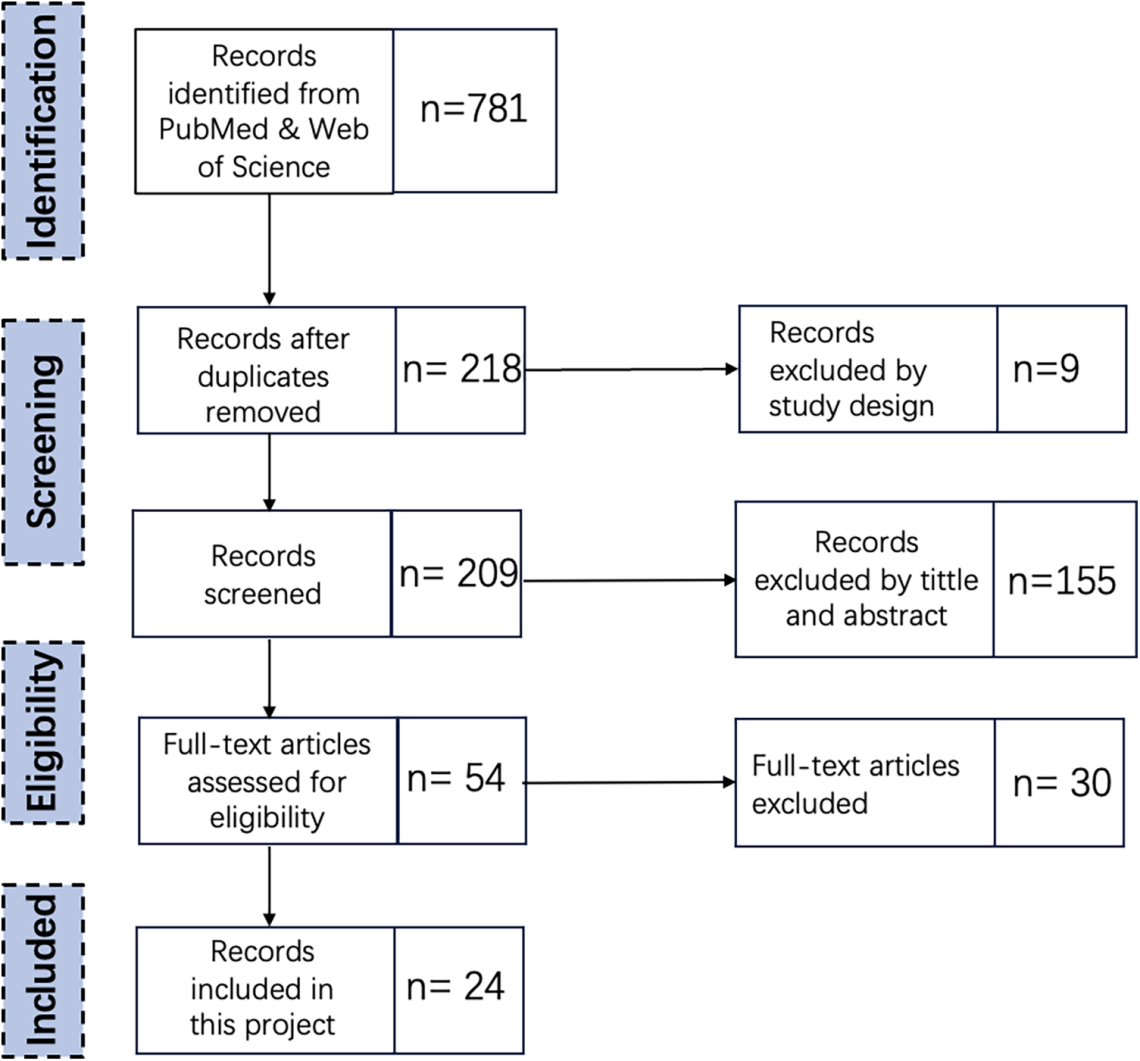
First round SLR appraisal steps.

A systematic literature review (SLR) methodology is employed to rigorously search (25–27). The process of SLR encompassed the following steps: Defining the research questions, determining the search strategy, screening titles and abstracts, then full-text review, quality assessment, data extraction and synthesis of results. Figure 1 shows the steps taken in the assessment process. To ensure coherence and consistency when selecting articles. It addresses the research questions: What AI strategies apply to Emergency Departments? Moreover, what challenges and opportunities are involved in using AI equipment in Emergency Departments?

**Figure 2:**
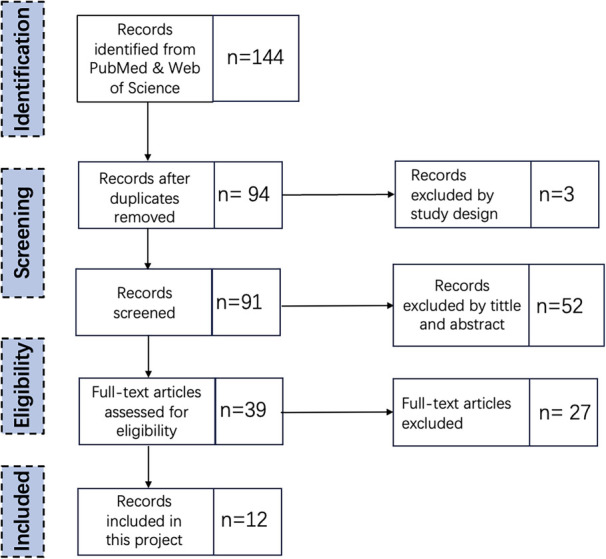
Second round SLR appraisal steps.

Figure 2 shows the steps taken in the appraisal process. This SLR addresses the research questions: What AI sensors can be utilised in Emergency Departments, and how do these AI sensors influence and transform the spatial design of Emergency Departments?

Detailed SLR methodologies are contained in Appendixes 1, 2. For the PICOS tables of the two rounds of systematic literature reviews, please refer to Tables 1 and 2.

**Table 1:** Picos table for first systematic literature review round.

	Inclusion criteria
Population	• Emergency Department patient • Emergency Department staff: Healthcare professionals, including doctors, nurses, and support staff working in emergency departments.
Intervention	Any intervention that included the use of AI-driven hardware and software tools, aimed at enhancing emergency medical services.
Comparator	Any comparison examining the state of emergency department care with and without the AI technologies.
Outcomes	• Patient-related Outcomes: Changes in patient experiences and treatment outcomes, including stress levels, treatment efficiency, and overall satisfaction with care received. • Staff-related Outcomes: Effects on emergency department staff, such as decision-making processes, healthcare delivery performance, workload, data collection efficiency, care efficiency, and cost-effectiveness.
Study design	Any primary studies presenting original research, with a clear methodology and results related to the application of AI in emergency departments.

**Table 2:** PICOS table for second systematic literature review round.

	Inclusion criteria
Population	Emergency departments: This includes both the physical spaces within hospitals where emergency care is provided and the staff and patients who interact within these environments.
Intervention	Any intervention that included the use of AI sensors directly in the design and operation of emergency departments.
Comparator	Examining the emergency departments with and without AI Sensor integration: Comparing the efficiency, effectiveness, and spatial design of emergency departments before and after the implementation of AI sensors.
Outcomes	• Impact on Emergency Department Design: Changes in the spatial layout, patient flow, and overall design of emergency departments resulting from the incorporation of AI sensors. • Operational Efficiency and Patient Care: Improvements in operational processes, patient triage, classification, and clinical management due to the integration of AI sensors. • Staff Perception and Job Design: Changes in medical staff's perception of their work environment, job satisfaction, and job design stemming from the use of AI sensors.
Study design	Any primary studies presenting original research, with a clear methodology and results related to the application of AI sensor in emergency departments.

## 5 Bibliographic analysis of emergency departments AI challenges field

This section presents the result of the bibliographic review into the challenges of AI use in Emergency Departments. In the first round of SLR, 24 articles were selected. The journals with the highest number of publications chosen were Sensors (3 articles), Journal of Ultrasound in Medicine (2 articles), and Frontiers in Cardiovascular Medicine (2 articles).

**Figure 3:**
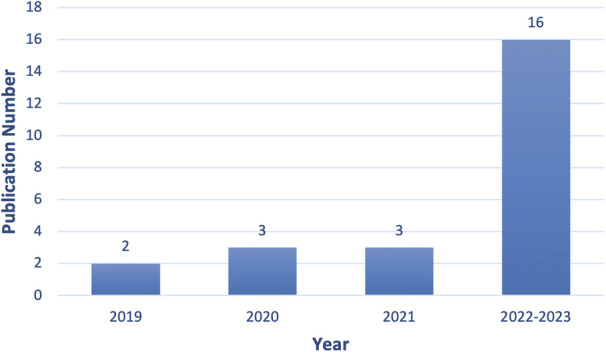
The number of publications since 2019 for the first SLR.

Between 2019 and 2023 (up to 1 July 2023), there has been a growing number of publications describing ED AI applications, as illustrated in Figure 3. In 2019, two articles were published. In 2020 and 2021, there were three papers. In 2022–2023, this escalated to sixteen.

Most of the 21 studies related to “Assisting Clinical Decision Support,” with 12 papers. Five publications extensively investigate the capacity of AI to predict cardiac events such as myocardial infarctions, cardiac arrests and heart failure. Each of “Assisting Medical Staff Operation”, “Patient Triage/Classification” and “Assisting Clinical Management/Influence on Job Design” are represented by 12.5% of the papers (Table 3).

**Table 3:** Number of publications by AI strategy.

AI strategies	Number of publications
Assisting clinical decision support	12
Prediction of myocardial infarction/cardiac arrest/heart failure	5
Assisting medical staff operation	3
Patient triage/classification	3
Assisting clinical management/influence on job design	3
Prediction of acute coronary syndrome/carotid disease	2
Prediction of vital signs	2
Prediction/identification of brain injury	1
Prediction of pulmonary embolism risk	1
Medical staff perception	1

The specific definitions of these AI strategies are presented in Appendix 3. Table 4 shows the various challenges associated with implementing Emergency Department AI.

**Table 4:** AI studies that defined ED implementation challenge.

Challenges	Studies on these challenges
Data quality limitations	(28–33)
Physical equipment and ED environment constraints	(30, 34)
Training and capability development issues	(31, 35–39)
High cost of implementation	(29)
AI technological constraints	(37, 38, 40, 41)

Each challenge is now elaborated.

### 5.1 Data quality limitations

In an examination of the 24 studies, 25% (*n* = 6) indicated that the principal challenge encountered by AI pertains to data-related issues. In Emergency Departments, patients often present in a state of stress. Under this stress and anxiety, the quality and accuracy of the data recorded might be compromised, subsequently affecting the performance of AI models (28). Similarly, AI heavily relies on substantial quantities of high-quality data (29). However, certain vital signs that necessitate manual collection can lead to data omissions or inaccuracies, impairing the functionality of AI devices (30). In the context of medical imaging, AI apparatuses typically demonstrate sensitivity to image quality. For instance, as is noted in (32), uncontrolled patient movements can degrade the quality of CT images. This poses a particularly daunting challenge in EDs, frequently demanding swift decision-making and actions. For certain conditions in emergency scenarios, there is an imperative for AI to gather and annotate diverse data to assist in diagnosis. For instance, AI models necessitate diverse datasets to accurately predict and detect various shock types—hemorrhagic, cardiogenic, neurogenic and septic—(33). However, collection and annotation of such diversified data can be challenging, especially for conditions that are either rare or elusive to diagnose.

### 5.2 Physical equipment and emergency departments environment constraints

The mobility of many medical devices, including those with embedded AI, presents a challenge in dynamic and fast-paced environments such as Emergency Departments. Equipping all patients with standard monitoring devices simultaneously might be challenging during peak times and emergencies due to financial constraints or routine demands (30). Similarly, various restrictions are imposed on AI (34). For instance, current AI-based clinical decision support tools frequently employ subjective criteria and overlook known risk factors or risk modulators, such as in cases of clinical pulmonary embolism. This might lead clinicians to order potentially unnecessary CT imaging, thus resulting in overutilisation and increased medical expenses.

### 5.3 Training and capability development issues

Utilising AI systems or devices necessitates a certain level of domain-specific and technical expertise. This involves understanding how to operate the system and grasping its fundamental principles and limitations. These requirements can constrain the utility of AI in emergency medicine, as not all emergency medical personnel are trained or proficient in harnessing such AI tools (37, 39). Likewise, some emergency physicians might not interpret specific medical images or data (e.g., chest radiographs) as accurately as a radiology specialist would. Effective deployment of AI tools still demands a degree of both medical and technical expertise. Misunderstandings concerning AI outputs could lead to misdiagnoses (36).

### 5.4 High cost of implementation

Implementing AI technologies in Emergency Departments entails significant costs, which might hinder their implementation (29). These costs include initial expenses associated with developing and acquiring new technologies, implementation costs such as modifications to existing infrastructure and ongoing maintenance and upgrading expenses. AI application will introduce indirect costs, such as staffing needs and personnel training.

### 5.5 AI technological constraints

While AI has many potential applications, certain technologies may have limitations that hinder utilisation in Emergency Departments (37, 40). For instance, technologies like Faraday modulation spectroscopy and cavity ring-down spectroscopy (CRDS) used for molecular detection are intricate and might only apply to specific types of gases (40). Although AI has the potential to assist in interpreting results from these technologies, their inherent complexity and specificity could limit their deployment in the fast-paced, broad-ranging environment of Emergency Departments. AI systems may need help considering and elucidating evidence effectively in scenarios demanding intricate diagnoses and often involving myriad factors and symptoms (41).

## 6 Bibliographic analysis of emergency department AI opportunities field

This section presents the result of the bibliographic review of the opportunities for AI use in Emergency Departments. In the second round of the systematic literature review (SLR), 12 articles were selected. The journals with the most chosen publications were Sensors (4 articles), Critical Care and Resuscitation (3 articles), BMC Emergency Medicine, International Journal of Emergency Medicine, PLOS ONE, Trials, and Journal of Clinical Monitoring and Computing, each with 1 article.

**Figure 4:**
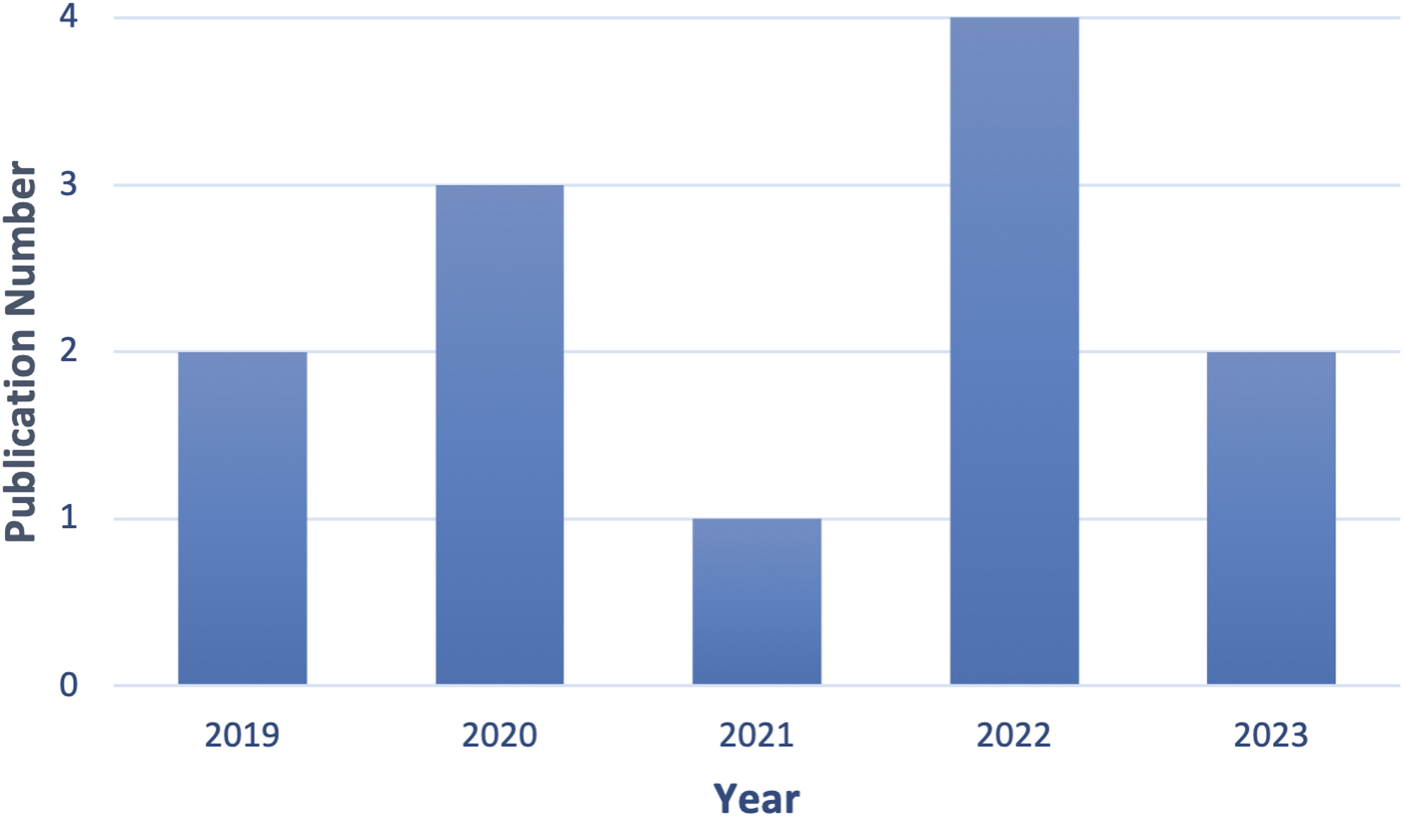
The number of publications since 2019 for the second SLR.

As Figure 4 shows, 2 of these articles were published in 2019, 3 in 2020, and the number grew to 4 in 2022. Fifty per cent of papers concerning AI sensors are directly related to Emergency Department design.

Table 5 shows the various opportunities associated with implementing AI in Emergency Departments.

**Table 5:** Studies of AI on opportunities.

Opportunities	Studies on opportunities
Support clinical decisions	(28, 30, 31, 33–36, 40–44)
Enhance healthcare professional performance	(28, 30, 33, 36, 38–41, 43, 45–47)
Reduce workload of clinicians	(28, 31, 32, 35, 37, 42, 45, 48)
Improve data acquisition	(29–32, 35, 40, 42)
Enhance efficiency (including cost)	(29–33, 37–41, 43, 45, 47, 49, 50)
Help in resource management	(47–49)

Each opportunity is now elaborated.

### 6.1 Supporting clinical decisions

AI has the potential to bolster clinical decision-making in emergency medicine by enhancing the speed, accuracy and efficiency of patient care. AI can provide early alerts and monitor vital signs and other patient data to anticipate adverse events, facilitating the timely identification of severe health conditions (28, 33, 42). Concurrently, learning using AI models can augment the precision and efficiency of diagnoses and prognoses for emergency personnel (34, 40, 43). An example is the pulmonary embolism result forecast model (PERFORM), which uses high patient data volumes to predict health outcomes. By furnishing patient-specific risk scores, these models can assist healthcare professionals in making more informed judgements. Some deep learning algorithms can identify vital anatomical structures, such as blood vessels, faster and more accurately than human experts. Such capabilities can elevate the speed and precision of diagnoses (35, 46). Furthermore, AI can aid physicians in diagnosing complex diseases based on a broader array of symptoms (41, 44), such as arteriosclerosis and fractures.

### 6.2 Enhance healthcare professional performance

In Emergency Departments' high-pressure, fast-paced environment, AI can automate repetitive tasks, minimise human errors and enable healthcare professionals to focus on the more intricate aspects of patient care (28). AI based on deep learning can outperform individual readers and radiologists in interpreting medical imaging, such as chest radiographs, leading to improved diagnosis accuracy and speed (36). AI can also aid in mitigating safety risks stemming from limited emergency resources by optimising resource allocation (45, 47). Furthermore, it can precisely predict clinical deterioration and swiftly detect critical conditions like shock and arteriosclerosis. It enables quicker interventions to enhance patient outcomes and reduce the time needed to address emergencies (30, 33, 39–41). AI can guide medical procedures, making them safer, swifter and more effective, especially for less experienced healthcare providers (30).

### 6.3 Reduce the workload of clinicians

AI can potentially alleviate the workload of clinical physicians in Emergency Departments through various mechanisms. AI can streamline tasks for doctors, reducing the time and expertise required by emergency physicians (35, 45). For instance, AI algorithms can assist in identifying blood vessels, thus enhancing peripheral vascular access for drug administration, laboratory tests and fluid infusion. Additionally, advanced AI systems can autonomously monitor patients, gather crucial information and suggest actions to physicians. This can decrease emergency doctors' time on patient monitoring and data interpretation and reduce the risk of human error (28, 42). AI can automate testing processes and offer specialised diagnoses. For example, once trained, AI systems can deliver diagnoses at the cardiology expert level, decreasing the need for on-site specialist expertise. By autonomously assessing image quality and accurately interpreting echocardiograms, AI can aid in completing medical imaging tasks such as disease detection and image reconstruction. This can ease the workload of radiologists and cardiologists while allowing broader provision of these services in settings where emergency specialists are limited (32, 37).

### 6.4 Improve data acquisition

Implementing AI in Emergency Departments can significantly enhance the data collection in multiple respects. AI algorithms, known for their robustness and adaptability, can effectively analyse data from various device types (35). Moreover, multi-agent systems exhibit adaptability in evolving situations, enhancing their utility and reliability even when input data are incomplete (42). Digital medical tools combined with AI technologies can rapidly and effortlessly gather and analyse vast medical data. This enables emergency physicians to receive timely decision insights, improving patient treatment outcomes (29, 31, 40). AI can address domain adaptation issues. For example, an AI solution trained on one type of ultrasound machine can be optimised to function effectively on different machine types, thus bolstering its applicability and efficiency (35). It can also bridge potential monitoring gaps arising from limited bedside access by healthcare professionals, ensuring continuous and comprehensive data collection (30). AI can correct motion artefacts in medical imaging without rescanning or employing additional systems, thus enhancing data quality and diagnostic accuracy (32).

### 6.5 Enhance efficiency (including cost)

AI can potentially enhance care efficiency and cost-effectiveness in Emergency Departments. By deploying remote monitoring, AI can make critical care services more accessible and efficient, potentially reducing patients' emergency department admission times and possibly decreasing mortality rates (29). AI can efficiently assist emergency personnel with automated triage in mass casualty incidents. This enables rapid categorisation of patients based on the severity of their symptoms, thus optimising resource allocation and potentially saving lives (49). AI can optimise chest pain triage and reduce door-to-balloon (D2B) times, which is crucial for treating heart attack patients. This can enhance patient outcomes and elevate Emergency Department efficiency (37). AI can accurately predict clinical deteriorations, thus shortening the prediction time for septic shock and diagnosing myocardial infarctions. Early predictions promote timely interventions, improving patient outcomes and decreasing care costs and complexity (30, 31, 33, 40, 41, 47).

### 6.6 Help in resource management

AI can be used in resource management in Emergency Departments. It promotes efficient reallocation of medical supplies and can analyse and predict varying supply usage rates and resource demands, thus assisting in allocation decisions (48). AI plays a pivotal role in more accurately assessing mass casualty incidents. By transmitting real-time on-site information to control centres, AI can provide up-to-the-minute situational updates and guide decisions related to resource allocation, personnel deployment, and crisis response strategies. This allows for more informed and targeted responses, potentially saving lives and alleviating the burden on emergency medical services (49). Machine learning algorithms can enhance understanding of disease progression by analysing patterns in patients' chest x-rays. This aids in predicting which patients are most likely to require ventilation and when and facilitates prioritising ventilator allocation and planning for future resource demands, potentially improving patient outcomes (47).

## 7 Integrating AI-sensors into emergency department design

The second round of the SLR identified 12 articles. The journal Sensors had the highest number of publications, with four articles, while eight other journals each contributed one article. Table 6 shows the various AI sensors used in Emergency Departments.

**Table 6:** AI-sensor studies direct to ED design.

AI strategies	Studies
Brain injury	(51)
Cardiac arrest and the ED	(52–54)
Clinical decision, management and physician stress	(55–57)
Vital signs	(30, 58–60)
ED patients triage	(61)

AI sensors were applied in two principal categories: strategies directly applied to Emergency Department designs and those used indirectly (such as wearable devices). While this second category might not directly influence design, data and insights can offer valuable perspectives for learning. Therefore, to provide a comprehensive view, both types of strategy were chosen to be examined in the scope of the systematic review. Each AI sensor is now elaborated.

### 7.1 Brain injury AI-sensors

For brain injury, as the Department of Intensive Care Medicine et al. noted, placement of a series of monitoring instruments and software, including the ICM+ neuro-monitoring software, at the patient's bedside allows real-time collection and analysis of high-resolution patient monitoring data (51). Bedside placement of equipment ensures that healthcare professionals can intervene promptly and base their actions on the changing physiological state of the patient, thus enhancing treatment outcomes.

### 7.2 Cardiac arrest AI-sensors

Regarding cardiac arrest treatment in the Emergency Department, Gould et al. discuss implementing ZOLL Medical's AccuVent™ technology in the design of Emergency Departments, particularly in resuscitation scenarios (52). This technology provides medical professionals real-time feedback on ventilation quality during resuscitation, allowing optimised decision-making and improved patient care. The information provided by the AccuVent™ system is displayed on defibrillators and monitors in numerical and graphic formats, and the system needs to be placed where healthcare personnel can quickly and easily view it and act accordingly. Setting monitors beside the patient enables medical professionals to access and read data easily.

Regarding cardiac arrest in Emergency Departments specifically, integrating wearable sensors can, to some extent, streamline the design of Emergency Departments, such as capacitive pressure sensors used during cardiopulmonary resuscitation (CPR) and feedback sensors integrated into a corpuls3® defibrillator. A capacitive pressure sensor, directly attached to an individual, enhances the accuracy and efficacy of chest compressions during CPR. This not only improves space utilisation but also influences the workflow of healthcare professionals, potentially making task execution smoother and with fewer constraints. On the other hand, the feedback sensor of the corpuls3® defibrillator placed on the patient's sternum needs easy access to the patient's chest area. An efficient electrical layout ensures continuous operation, and optimal positioning of the defibrillator facilitates real-time monitoring without impeding the workflow (53, 54).

### 7.3 Clinical decisions, management and physician stress AI-sensors

Sensors measuring the cognitive load on physicians can track and quantify psychological and mental stress in doctors when managing patient situations. This can aid in timely task distribution and workload adjustments to prevent decision-making errors. Additionally, by analysing this data, doctors can better understand and optimise their decision-making processes, thus enhancing diagnostic accuracy and treatment effectiveness. Kennedy-Metz et al. discuss how sensors assessing physicians' cognitive load, including heart rate sensors and near-infrared spectroscopy (NIRS) sensors, are studied (55). The Polar H10 heart rate sensor is a wireless device worn by doctors on an adjustable elastic chest strap which captures and transmits all heart rate data wirelessly. However, the NIRS sensor—Medtronic's INVOS™ 5,100C cerebral/somatic oximeter used for collecting predicted rSO2 values in the bilateral prefrontal cortex areas—requires a wired connection. Consequently, the spatial design of the Emergency Department must allow the preamplifier to be placed close to the monitored doctor and adjust its position as the doctor moves around the operating table. Moreover, the INVOS™ monitor must be placed near the preamplifier and on top of the cardiopulmonary bypass pump.

Within the Emergency Department setting (56), they discussed using a portable OpenICE monitor. This collects electrocardiogram (ECG) data to monitor clinical team members' heart rate variability (HRV). The system evaluates the cognitive load on clinicians in real time. It provides real-time feedback and alerts based on changes in cognitive load, thus strengthening clinical management, promoting effective team coordination and assisting in making more informed clinical decisions (57). introduces the Healthdot sensor, which improves patient care in an acute admissions ward by continuously assessing patient vital signs. This wearable sensor is mounted on the patient's rib, on the left midclavicular line of the lower abdomen, thus facilitating patient mobility and potentially alleviating caregivers’ workload. Incorporating this sensor in Emergency Department design necessitates considering patient beds and furniture that can accommodate the installation of the sensor and adapt to the patient's movement.

### 7.4 Patient vital signs monitoring

Some compact wireless single-sensor monitoring devices must be used with a display (58). It can continuously measure blood pressure, pulse, respiration, and oxygen saturation. This noninvasive device employs photoplethysmographic technology and consists of an emitter, a sensor and a charging cable. Monitors receiving data from the device via Bluetooth must be placed in locations easily visible and accessible by healthcare professionals. Similarly, Satake et al. also necessitate using a sensor with a display (59). A noninvasive continuous arterial pressure monitoring device utilising micro-electro-mechanical systems (MEMS) technology can be applied in high-risk settings like Emergency Departments and intensive care units where continuous blood pressure monitoring is crucial. The MEMS device presses a sensing membrane directly onto the skin above the radial artery. It transmits the voltage measured to the monitor display with a signal amplification circuit. This necessitates careful analysis of sightlines and potential obstructions in the emergency room to ensure that doctors can easily view the screen from various angles, enabling them to assess the patient's condition and make corresponding decisions swiftly.

CCTV can also be utilised in emergency department intensive care units (ICUs) to monitor vital signs (60). Employed CCTV cameras in an ICU for continuous cardiopulmonary monitoring. These cameras utilised a camera-based photoplethysmography method to remotely measure subtle colour changes in the skin caused by arterial pulsations, which allowed non-contact heart rate measurement. Additionally, they measured the movement of the intercostal muscles in the chest and abdomen during inhalation and exhalation, translating it into a respiration rate. The CCTV cameras need to be mounted on the ceiling and positioned to face the patient, with the upper half of the patient centred within the video frame. This necessitates an unobstructed clear view of the patient from above and strategically placing any equipment, lighting and other architectural elements to prevent any obstruction in the camera's line of sight.

Hicardi sensors have been used to monitor patients in Emergency Departments continuously. The sensor attempts to foster early detection and prediction of clinical deterioration in patients at risk of septic shock, especially those presenting with stable febrile conditions. The Hicardi sensor monitors single-lead electrocardiograms, the respiratory rate, skin surface temperature, and patient position and activity. Adhering to a patient's chest facilitates uninterrupted data collection for up to 24 h. Consideration must be given to storage and charging solutions for Hicardi devices. These devices should be placed in easily accessible locations near the triage or admission areas to ensure swift deployment.

### 7.5 Emergency department patients triage

Innovative approaches to assessing patients in Emergency Departments have included incorporating a wearable triage system. This integrated sensor system is designed to wirelessly monitor patients' respiratory and cardiac activities. This enhances the efficiency and effectiveness of the triage process and reduces the workload of triage nurses.

## 8 Discussion

### 8.1 AI-sensor informed ED design and flow

An array of challenges and pressures confronts Emergency Departments. These challenges emanate not only from the complexity and diversity of patients but also from the grave repercussions of acute injuries, violent behaviour and public safety incidents. Various strategies apply AI in emergency settings that will affect medical staff workflows, behaviour and patient needs. In traditional emergency room designs, emphasis is placed on space utilisation, circulation and intuitiveness (62). However, with the widespread application of AI sensors, designers should reconsider the functionality and adaptability of emergency rooms. For instance, in cases of critical care such as brain injuries, to achieve real-time monitoring and immediate access to a patient's physiological parameters, there is a need for precise arrangement of beds and the placement of related equipment. What is certain is that understanding the cognitive load on doctors (mediated by AI sensors) becomes even more paramount. While some sensors offer wireless flexibility, others necessitate fixed wired connections. These design considerations directly impact the space dynamics and enable areas in the Emergency Department to adapt and evolve based on varying technological requirements.

Moreover, the rise in wireless monitoring tools and the pivotal role of display monitors in modern healthcare underscore the importance of visibility in Emergency Department design. To allow medical professionals instant access to patient data, the positioning and accessibility of data display screens and other interactive interfaces become particularly crucial. The design must consider optimal sight lines and ease of operation for displays to ensure professionals can obtain the necessary information in urgent situations. Innovations like the integration of CCTV for vital sign detection in ED-ICU environments further demonstrate how architectural design can either facilitate or hinder AI technology. For instance, CCTV cameras require strategically unobstructed positions, which guide architectural decisions related to ceiling design, lighting placement and other overhead infrastructure elements.

Integration of AI presents novel demands compared to conventional architectural design strategies. To effectively incorporate AI technologies, the design of Emergency Departments no longer solely focuses on space utilisation, circulation and intuitiveness. Assimilation of AI demands higher levels of functionality and adaptability. This involves the technology and how medical professionals interact with these tools, access and interpret data, and enhance the quality and efficiency of patient care. These new sensors may, however, increase the cognitive load on physicians. Therefore, the design of emergency rooms must minimise additional burdens while ensuring doctors can swiftly and effortlessly access and utilise the necessary tools and information.

### 8.2 Considering AI sensor use in a resuscitation unit in China

**Figure 5:**
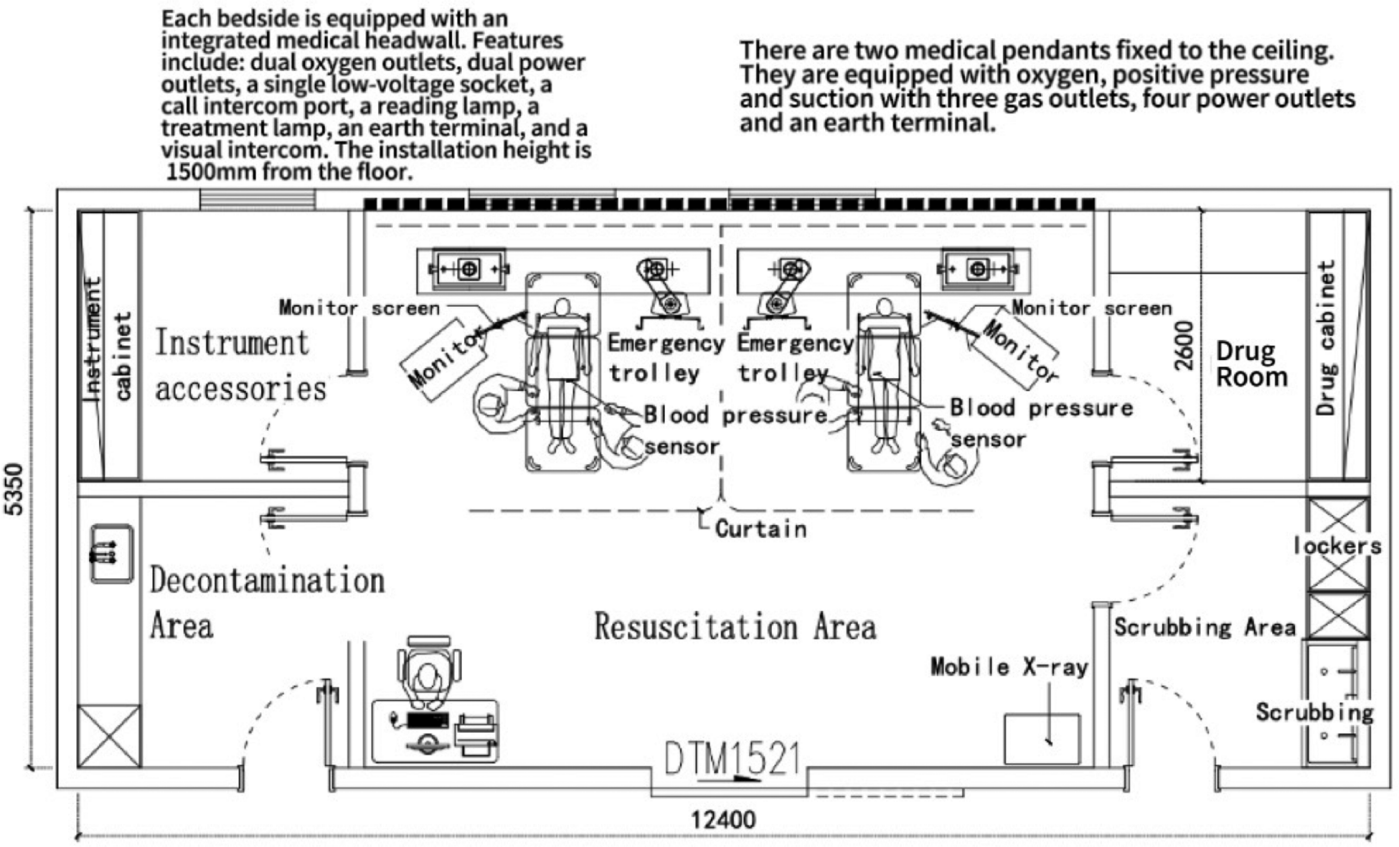
Optimised Resuscitation Unit.

Building on the Chinese layout of the Resuscitation Unit in the “Modern Hospital Building Design Reference Album” by Zhang (63), Figure 5 presents a possible AI sensor-enabled Unit layout. This arrangement may enhance cardiopulmonary resuscitation (CPR) efficiency, effectiveness, real-time ventilation quality feedback, and monitoring of blood pressure. Integration of these real-time feedback sensors could give instantaneous feedback during the CPR process, allowing the medical team to swiftly adjust and respond to varying circumstances.

Both over-ventilation and insufficient ventilation can be detrimental to patients. Overly rapid ventilation may lead to air entering the stomach, which consequently increases the risk of vomiting and decreases the likelihood of successful resuscitation. Ventilation quality sensors can monitor the actual ventilation volume and compare it with the recommended range, thus assisting healthcare professionals in adjusting their techniques. In cases of airway obstruction, these sensors detect restricted airflow and signal to healthcare providers that they need to clear the airway or change the position of the head and chin. These ventilation quality sensors are positioned between the ventilation bag and the patient's airway. They continuously monitor the respiratory rate and ventilation volume, ensuring precision and stability throughout resuscitation.

When a patient's heartbeat returns during CPR, a rise in blood pressure is often one of the earliest indicators. Blood pressure sensors can assist healthcare professionals in promptly detecting and confirming the restoration of a heartbeat. Moreover, these sensors can continuously monitor blood pressure, aiding the evaluation of the effectiveness of resuscitation. This continuous feedback allows physicians to adjust the depth and frequency of compressions to achieve optimal blood circulation.

Installing high-definition monitors compatible with both types of sensors is essential to facilitate the medical team's real-time observation of these critical data.

The position of the high-definition monitors has been decided considering the practical needs of the medical team during operations. Positioning the monitors at the side of the patient's bed ensures that healthcare professionals can view the vital data from all angles. Additionally, an advanced medical headwall system is installed for each bed space.

### 8.3 Considering AI sensor use in an emergency operating room in China

**Figure 6:**
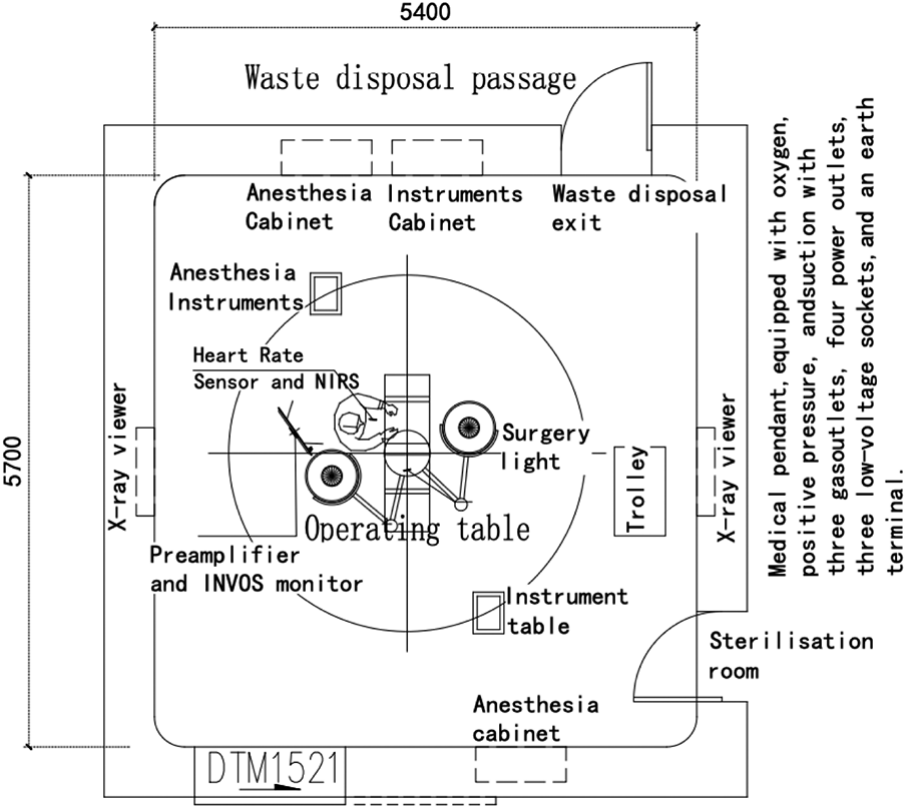
Optimised OR.

Figure 6 depicts the design of an emergency operating room (OR) adapted from Zhang (63) to include AI sensors for use in China. Emergency OR is an approach not widely used in countries such as the United Kingdom but has historically been seen. Monitoring the physical and psychological states of the surgeon may contribute to knowledge of the progression and cognitive load of surgical procedures. It could provide alerts of potential excessive fatigue or heightened stress levels. A heart rate sensor and a near-infrared spectroscopy (NIRS) sensor could be combined. The heart rate sensor, attached to an elastic chest strap, provides real-time monitoring of the doctor's heart rate, offering a preliminary assessment of their psychological state. The NIRS sensor on the doctor's forehead measures brain oxygenation and blood flow changes, furnishing further insights into the doctor's cognitive state. However, the NIRS sensor, which may require a wired connection, could impede the doctor's surgical movements.

An accompanying preamplifier and INVOS™ monitor could receive and process data from the NIRS sensor. Putting sensors behind the doctor's back could ensure continuity of data transmission, smooth surgical operations and avoid the potential inconveniences of a wired connection.

### 8.4 Considering AI sensor use in the emergency department ICU in China

**Figure 7:**
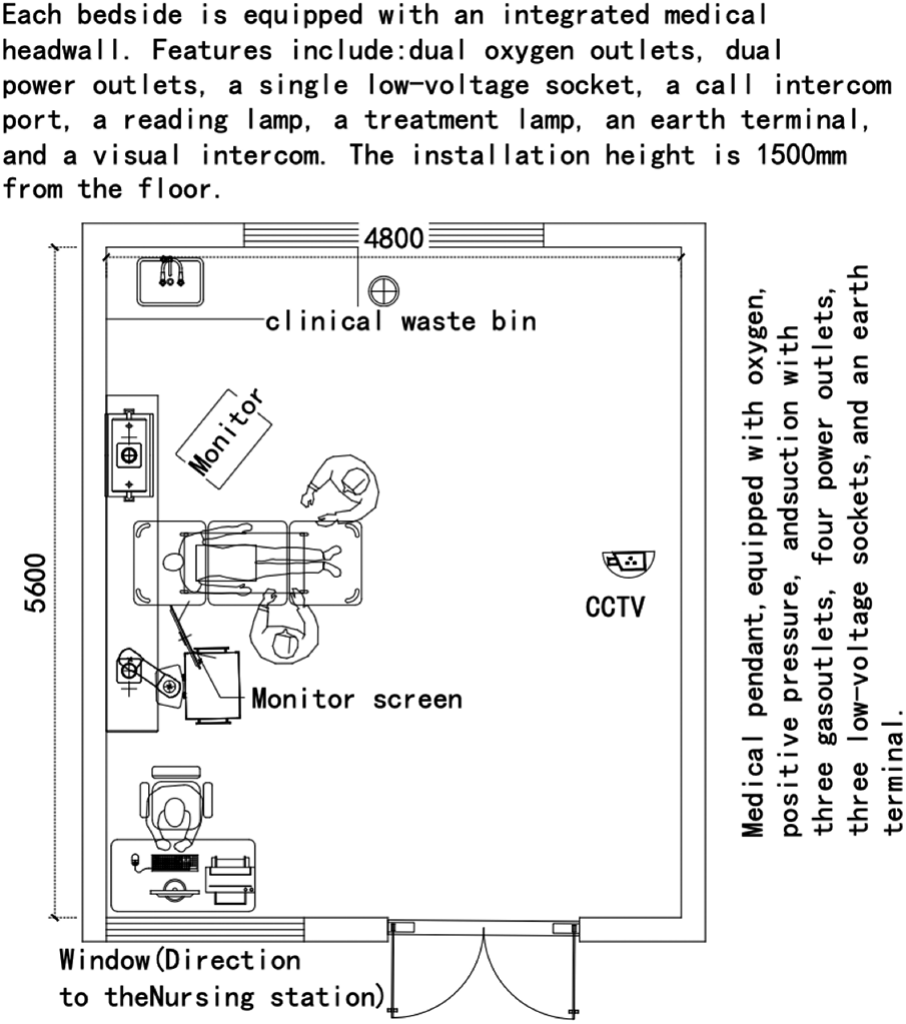
Optimised ED ICU.

Figure 7 shows an Emergency Department ICU from Zhang (63), adapted to include AI sensors in China. This could enhance the quality of patient monitoring and the responsiveness of healthcare professionals. A continuous blood pressure monitoring device and a CCTV system are installed. A blood pressure monitoring device could give the medical team continuous real-time blood pressure readings. This immediate feedback could reveal early signs of patient deterioration, such as shock, heart failure, cerebral haemorrhage or other complications. After identifying these signs, healthcare professionals can quickly respond and implement necessary therapeutic measures. Furthermore, this continuous blood pressure monitoring also aids healthcare professionals in assessing the effectiveness of treatment protocols, whether pharmacological treatments or mechanical ventilation.

Introducing CCTV provides non-contact continuous cardiopulmonary monitoring in the Emergency Department ICU. These cameras could utilise advanced photoplethysmographic techniques, remotely measure minute skin colour changes caused by arterial pulsations, and enable non-contact heart rate measurement. Simultaneously, the CCTV system can calculate respiratory rates by capturing movements of the intercostal muscles between the chest and abdomen during inhalation and exhalation (60). However, to ensure the accuracy and reliability of the CCTV system, the camera must have a clear, unobstructed overhead view of the patient. Therefore, when configuring the ICU space, it is essential to strategically position equipment, lighting and other structural elements to ensure the camera's field of view remains unobstructed, thus guaranteeing optimal performance.

## 9 Conclusion

The literature on AI-enabled Emergency Department design is limited. There is literature that explores how Emergency Departments can be designed to use sensors and literature on AI-informed sensor design, but the two are not strongly connected. This study has explored the connection through a two-stage literature review on AI-informed sensor design and sensor implementation in Emergency Department design. What has been shown is the importance of innovation in Emergency Department design. Monitoring vital signs, quickly identifying cardiac arrest, and clinical decision strategies are critical. An analysis of standard Chinese hospital Emergency Department design of the Modern Hospital Building Design Reference Album (63) showed that there could be a more advanced and innovative approach to AI design in Chinese resuscitation units, Emergency Department operating rooms and the Emergency Department intensive care units.

It was evident that AI technologies offer significant advantages in emergency clinical decision-making, resource optimisation and patient monitoring. These technologies could enhance the accuracy of diagnosis and aid medical professionals in a high-pressure environment, thus boosting the overall efficiency of medical care. However, applying AI in emergency settings also faces numerous challenges. Issues related to data quality, significant cost implications, the need for specialised operational skills and potential misdiagnoses might impede the widespread adoption of AI in Emergency Departments.

The relationship between AI sensor design and the architectural planning of Emergency Departments needs further consideration. AI sensor specification guides could help adoption in the design of Emergency Departments. Considering bed arrangement and the positioning of data display screens could ensure a harmonious blend between technology and practical workflow. AI sensors could enhance nursing efficiency, ensure patient safety and amplify the operational capabilities of medical personnel. Sensors can provide real-time feedback and precision during cardiopulmonary resuscitation procedures in the resuscitation unit. Physicians' physiological and psychological conditions can be incorporated into the emergency operating room to predict and mitigate potential issues related to excessive fatigue or stress. Meanwhile, in the Emergency Department ICU, advanced sensor monitoring systems may ensure the continuity and efficiency of patient care. Integrating AI technologies creates opportunities to refine Emergency Departments' design and operational modalities further, offering a more efficient and safe environment for medical staff and patients.

## 10 Limitations

This study has used a systematic literature review as the primary research methodology. While this provided a comprehensive understanding of existing research, it may also result in the omission of practice-based studies available in grey literature or unpublished studies. Empirical research is needed, as is feedback directly obtained from frontline healthcare workers, patients and other relevant practitioners. There was a geographical focus on China so that other healthcare systems would vary. There will be differences in Emergency Department design and AI applications due, for example, to differences in culture, public health, healthcare system, primary care use, and technology adoption. Finally, Artificial Intelligence is a rapidly evolving field, and the emergence of new technologies and applications may make existing research quickly obsolete.

## Data Availability

The original contributions presented in the study are included in the article/Supplementary Material, further inquiries can be directed to the corresponding author.

## Appendix 1 - Methodology of the First Systematic Literature Review Round

### Research questions

1. What AI strategies are applicable to Emergency Departments according to the existing literature?
2. What challenges are involved in using AI equipment in Emergency Departments, as reported in the literature?
3. According to current research, what opportunities emerge for using AI equipment in Emergency Departments?

### Selecting search strings

Given that the first round primarily investigated the application of AI in Emergency Departments, the key terms employed were “ED” and “AI”. The AI-related keywords were “artificial intelligence equipment” and “artificial intelligence technology equipment.” The ED-related keywords were “Emergency Department,” “emergency services” and “emergency medicine.” A Boolean logic search was employed to ensure comprehensive and relevant results by strategically combining these terms.

### First round study selection

Inclusion Criteria:

(1) Type of studies: peer-reviewed articles and primary research studies.
(2) Language: studies written exclusively in English.
(3) Text availability: must be available in full text.
(4) Publication year: no earlier than 2019. Given the rapid advance of technologies like AI, literature that is more than five years old may be outdated.

Exclusion Criteria

(1) Type of study: books, editorials, opinion pieces, and studies in which AI is not directly used or relevant to Emergency Departments.
(2) Language: studies not written in English.

### Database search

For the initial round, two databases were selected: Web of Science and PubMed. Identical search terms and a specified time range (from 2019 to 2023) were used.

• In the Web of Science dataset, 169 articles were retrieved.
• In the PubMed database, 612 articles were retrieved.

Combining the results of the two searches, 781 relevant articles were identified. The literature search was completed on 1 July 2023.

### Appraisal steps in the systematic literature review

During the assessment phase, the selected articles were narrowed down to the most relevant ones. This stage involved article screening and quality assessment. The assessment procedure involved multiple iterations.

• First Iteration: The primary step involved removing duplicate papers. By importing the 781 articles into EndNote X9 and utilising its automatic deduplication feature, 563 redundant articles were discarded, leaving 218 unique articles.
• Second Iteration: This step involved scanning the titles and abstracts to exclude irrelevant articles. After this iteration, a refined list of 54 articles remained.

All 54 articles were read further in the quality assessment to gauge their relevance and eligibility for the analysis. This round of article screening was grounded on the quality criteria below.

### Quality assessment criteria

Q1: Clarity of research methodology and consistency between the method and its implementation.

The study should elucidate how AI was implemented in an emergency department, the type of AI employed (e.g., machine learning or natural language processing), and the specific problem it aimed to address.

Q2: Description of the devices, techniques and knowledge involved in the AI application.

The study should provide a clear and detailed account of the AI hardware and software used, the data sources and the types of algorithms.

Following this step, the first round of the SLR resulted in a final sample consisting of 24 articles.

## Appendix 2 - Methodology of the Second Systematic Literature Review Round

### Research questions

1. What are the potential applications of different types of AI sensors in the design of Emergency Departments?
2. How do these AI sensors influence and transform the spatial design of Emergency Departments?

### Selection of search strings

In this research phase, the keywords selected for the search were “AI strategies,” “ED design”, and “sensor.”

AI strategies: drawing on the findings of the first SLR, the keywords were “brain injury,” “myocardial infarction,” “cardiac arrest,” “heart failure,” “acute coronary syndrome,” “carotid disease,” “vital signs,” “pulmonary embolism,” “clinical decision,” “medical staff operation,” “patient triage,” “patient classification,” “clinical management,” “job design” and “medical staff perception.”

ED design: The keywords were “Emergency Department design” and “ED design.”

A Boolean logic search was employed to ensure comprehensive and relevant results by strategically combining these terms.

### Second round study selection

Inclusion Criteria

(1) Type of study: peer-reviewed articles and primary research studies.
(2) Language: studies written exclusively in English.
(3) Text availability: must be available in full text.
(4) Publication year: no earlier than 2019. Given the rapid advance of technologies like AI, literature that is more than five years old may be outdated.

Exclusion Criteria

(1) Type of study: books, editorials, opinion pieces and studies in which AI sensors are not involved in the design of Emergency Departments.
(2) Language: studies not written in English.

### Database search

In the second round of the review, the same Web of Science and PubMed databases were employed. The specified search terms and date range (from 2019 to 2023) were used.

• A total of 83 articles were retrieved from the Web of Science dataset.
• From the PubMed database, 61 articles were retrieved.

Combining the results of second-round searches, 144 relevant articles were identified. The literature search was completed on 27 July 2023.

### Systematic literature review appraisal steps

During the assessment phase, the selected articles were narrowed down to the most relevant ones. This stage involved article screening and quality assessment. The assessment procedure involved multiple iterations.

• First Iteration: The initial step involved removing duplicate papers. By importing the 144 articles into EndNote X9 and leveraging its automatic deduplication feature, 50 redundant articles were discarded, leaving 94 articles.
• Second Iteration: This step involved scanning the titles and abstracts to weed out irrelevant articles. After this iteration, a concise list of 39 articles was obtained.

For the quality assessment, each of the 39 articles was read to determine its relevance and eligibility for the subsequent analysis. The selection in this round was based on the quality criteria below.

### Quality assessment criteria

Q1: Clarity of Reasons for Using AI Sensors

The study should elucidate in detail how Emergency Departments employ AI sensors. For instance, whether the sensors are employed to monitor patients' vital signs or to improve patient flows, among other uses.

Q2: Detailed Explanation of the Equipment, Techniques and Knowledge Involved in the Application of AI Sensors.

The study should comprehensively and precisely describe the AI hardware and software used, the data sources and the type of algorithms.

After filtering using the above criteria, the second round of the SLR resulted in a final sample comprising 12 articles.

## Appendix 3 - AI Strategies

On reviewing the full content of the 24 articles, 10 AI strategies were identified:

(1) Prediction/Identification of Brain Injury: AI can predict or identify potential brain injuries. It typically works by analysing medical imaging or other diagnostic data, thus facilitating early diagnosis and intervention.
(2) Prediction of Myocardial Infarction/Cardiac Arrest/Heart Failure: The AI strategies focused on predicting cardiac events like myocardial infarction, cardiac arrest and heart failure. This is typically achieved by analysing patient data, including electrocardiograms and other relevant health indicators.
(3) Prediction of Acute Coronary Syndrome/Carotid Disease: AI forecasts the occurrence of acute coronary syndromes or carotid diseases, typically by analysing imaging results and other related metrics.
(4) Prediction of Vital Signs: AI predicts changes or abnormalities in vital signs such as the heart rate, blood pressure, body temperature and respiratory rate. This is generally based on real-time monitoring data.
(5) Prediction of Pulmonary Embolism Risk: AI analyses patient imaging data and other factors to assess the likelihood of a pulmonary embolism, aiding early detection and prevention.
(6) Assisting Clinical Decision Support: AI supports healthcare professionals in making clinical decisions by offering risk assessments, diagnostic suggestions and other insights based on comprehensive health data analysis.
(7) Assisting Medical Staff Operation: AI optimises medical staff operations, which may include assistance in procedures such as intubation.
(8) Patient Triage/Classification: AI helps determine patients' care priorities based on their health condition or the severity of their symptoms, ensuring that those in most urgent need receive timely attention.
(9) Assisting Clinical Management/Influence on Job Design: AI optimises clinical management practices, facilitating the rational planning of medical resources and minimising waste. This leads to better technology integration, enhancing patient care efficiency.
(10) Medical Staff Perception: Research in this category focuses on understanding how medical professionals perceive, accept and interact with AI. It explores how AI is employed in clinical settings and its implications for integration and effectiveness in patient care.
