# Supplementary material for: Designing Chinese hospital emergency departments to leverage artificial intelligence—a systematic literature review on the challenges and opportunities
Source: Front Med Technol. 2024 Mar 21;6:1307625. doi: 10.3389/fmedt.2024.1307625 (PMC10991761; doi:10.3389/fmedt.2024.1307625)
Supplement: Supplementary file 1 [file Table1.docx]

Supplementary Material

# Supplementary Tables

| Colour | AI strategies |
| --- | --- |
|  | Prediction/identification of brain injury |
|  | Prediction myocardial infarction/cardiac arrest/heart failure |
|  | Prediction of acute coronary syndrome/carotid disease |
|  | Prediction of vital signs |
|  | Prediction of pulmonary embolism risk |
|  | Assisting clinical decision support |
|  | Assisting medical staff operation |
|  | Patients triage/classification |
|  | Assisting clinical management/influence on job design |
|  | Medical Staff Perception |

Supplementary Table 1: Colour Coding of 10 AI Strategies

| Article | Colour | | | Main aim of study |
| --- | --- | --- | --- | --- |
| 1. (Al-Dmour et al., 2019) |  |  |  | Patient classification design |
| 1. (Banerjee et al., 2019) |  |  |  | Prediction of pulmonary embolism risk |
| 1. (Blaivas, Arntfield and White, 2020) |  |  |  | Peripheral venous catheter placement help for medical novices |
| 1. (Lanza, Seidita and Chella, 2020) |  |  |  | Assisting clinical decision support |
| 1. (Scott et al., 2020) |  |  |  | Increasing health care providers' capability and capacity |
| 1. (Bednarski, Singh and Jones, 2021) |  |  |  | Optimising the redistribution of critical medical supplies |
| 1. (Borisov et al., 2021) |  |  |  | Prediction of acute myocardial infarction |
| 1. (Kim et al., 2021) |  |  |  | Chest radiograph interpretation |
| 1. (Apiratwarakul et al., 2022) |  |  |  | Assessing numbers of patients |
| 1. (Bin et al., 2022) |  |  |  | Reducing non-essential activities to improve care efficiency |
| 1. (Blaivas, Blaivas and Tsung, 2022) |  |  |  | Assessing inferior vena case collapse |
| 1. (Chen et al., 2022) |  |  |  | Assisted detection of ST-elevation myocardial infarction |
| 1. (A. Choi et al., 2022a) |  |  |  | Predicting septic shock in febrile patients |
| 1. (J. Choi et al., 2022) |  |  |  | Tracheal intubation guide systems |
| 1. (Duanmu et al., 2022) |  |  |  | Prediction of mortality and length of time on invasive mechanical ventilation |
| 1. (Emakhu et al., 2022) |  |  |  | Acute coronary syndrome prediction |
| 1. (Hardalaç et al., 2022) |  |  |  | Wrist fracture detection |
| 1. (Jha et al., 2022) |  |  |  | Perceptions and knowledge of artificial intelligence in medicine |
| 1. (Lin et al., 2022) |  |  |  | Process echocardiograms, rapid triage of patients presenting with chest pain |
| 1. (Rajagopalan et al., 2022) |  |  |  | Delivering effective CPR |
| 1. (Yao et al., 2022) |  |  |  | Assessment of advanced treatment of heart failure |
| 1. (Zhou et al., 2022) |  |  |  | AI motion correction algorithms for cerebral CT |
| 1. (Arsenescu et al., 2023) |  |  |  | Carotid artery spatial orientation and evaluation results |
| 1. (Jeong et al., 2023) |  |  |  | Patient Shock Detection |

Supplementary Table 2: 24 Papers Coded based on AI Strategies

| Number | Author, Year &  Country | Number of participants | Study Aim | Findings about AI used in A&E |
| --- | --- | --- | --- | --- |
| 1. | Al-Dmour, J. A.  Sagahyroon, A.  Al-Ali, A. R.  Abusnana, S. (2019)  UAE | 34 | Designing a fuzzy logic-based patient warning system for deterioration | Challenges:  Uncertainty and imprecision in real-world data: AI systems need to effectively handle vagueness, imprecision, and uncertainties in real-world medical data.  Opportunities:  Aid in early detection of serious health conditions by monitoring vital signs and predicting adverse events.  Mimic human thought processes in complex scenarios, and can execute repetitive tasks efficiently, potentially improving the overall health care performance.  Enables remote health monitoring, helping cater to an aging population and allowing for healthcare to be more accessible.  Fuzzy logic excels in handling imprecise information and can make decisions based on approximate data, similar to human decision-making processes. |
| 2. | Banerjee, I., et al. (2019).  US | 3214 | Developing a machine learning model that generates patient-specific PE risk scores using longitudinal clinical data, intended to support clinical decisions for patients needing CT scans for PE. | Challenges:  Existing clinical decision support (CDS) tools have limitations, including the use of subjective criteria and the omission of known clinical pulmonary embolism (PE) risk factors or risk modifiers. This can lead clinicians to order CT imaging against guidelines, potentially leading to overuse and unnecessary healthcare costs.  Opportunities:  A machine learning modeling approach, such as the Pulmonary Embolism Result Forecast Model (PERFORM), can predict PE imaging outcomes based on a multitude of patient data, including demographics, vital signs, diagnoses, medications, and lab results. This model could provide a patient-specific risk score and may be more efficient and accurate than existing PE risk scoring systems. |
| 3. | Blaivas, M., et al. (2020).  US | 183,522 | To aid novices in identifying key anatomical structures in ultrasound images for upper extremity peripheral vascular access | Challenges:  Training of healthcare professionals: Using AI systems like the studied deep learning (DL) algorithm requires adequate training, which may be hard to achieve consistently due to nursing shortages and high turnover.  Opportunities:  The study's DL algorithm outperformed human experts in identifying key anatomical structures, suggesting that AI can enhance the speed and accuracy of diagnoses.  The algorithm can assist in identifying blood vessels, potentially improving peripheral vascular access for medication administration, laboratory testing, and fluid delivery.  The DL algorithm developed can label structures in real-time video, indicating its potential for immediate clinical use, such as aiding novice providers in finding cannulation targets.  Robustness and adaptability: Despite sourcing data from varied equipment, the algorithm was still effective, suggesting it may be robust and widely applicable across different types of ultrasound machines in real-world clinical settings. |
| 4. | Lanza, F., et al. (2020) |  | To develop a self-adapting, intelligent, multi-agent system for healthcare that can monitor patients and assist physicians in decision-making in complex clinical scenarios, using a robot as its deployment platform. | Opportunities:  AI and robotics could provide intelligent systems to support physicians in making decisions in dynamic contexts.  Multi-agent systems could adapt to changing situations, even in the absence of complete input data.  Intelligent systems could autonomously monitor patients, gather key information, and suggest actions to physicians.  The application of the agent-oriented paradigm and robotics could help in solving complex healthcare scenarios. |
| 5. | Scott, B. K., et al. (2020).  US |  | Aid patients while minimising the spread of COVID-19, enhance the ability and resources of health care providers, and anticipate and avert potential future outbreaks | Challenges:  Adoption barriers: The development time and high costs can be potential barriers to the adoption of new technologies, such as a telementoring system.  Data quality: The absence of clean and large amounts of data typically required for AI systems is a challenge.  Opportunities:  Telecritical care makes critical care services more available outside of the traditional ICU and more efficient within it. It can reduce the length of stays and lower patient mortality.  Telementoring can extend patients' access to clinical expertise even if they live in areas with limited providers and specialists.  Data analysis for decision-making: Digital health tools and technologies make it possible to collect and analyze data rapidly and easily.  Remote patient monitoring. |
| 6. | Bednarski, B. P., et al. (2021).  US |  | Explores how reinforcement and deep learning can optimise medical equipment redistribution, enhancing public health response to future crises like COVID-19 | Opportunities:  AI can facilitate efficient redistribution of medical supplies during public health crises.  AI models have the potential to handle complexity better and improve their performance as task complexity increases. |
| 7. | Borisov, A. V., et al. (2021).  Russia | Target group 32, control group 42 | Explore effective machine learning algorithms for constructing a predictive model to diagnose acute myocardial infarction (AMI) using exhaled air spectral data | Challenges:  The specificity of volatile markers present in patients' exhaled air is not high, which can lead to false positives or false negatives.  Techniques used for molecular detection, like Faraday modulation spectroscopy or Cavity Ring-Down Spectroscopy (CRDS), have limitations in terms of complexity, or are only applicable for certain types of gases.  Opportunities:  More accurate and faster diagnosis.  Volatile markers associated with AMI present a new avenue for diagnosing the disease.  Machine learning methods can be employed to create classification rules without explicit programming or expert evaluation.  Advanced detection methods like Laser Photo-acoustic Spectroscopy (LPAS) can provide improved sensitivity in molecular detection. |
| 8. | Kim, J. H., et al. (2021).  Korea | 388 | Investigate whether DLCR supports CR interpretation and the clinical decision-making of ED physicians | Challenges:  The interpretation of CR by emergency department physicians is not as accurate as that by radiology experts.  For critically ill patients who require fast CR interpretation, ambiguous results might be overlooked, leading to negative impacts on patient safety.  In rural areas, emergency care resources are limited and there is a lack of advanced imaging equipment like CT or MRI scanners.  Opportunities:  Deep learning-based auxiliary technology for CR interpretation is more accurate than interpretations made by individual readers or radiologists.  The use of DLCR can support emergency physicians in interpreting CR, especially those with limited experience or resources.  This technology could be effectively utilised in resource-scarce areas.  Algorithms for automatic detection have the potential to play a useful role in clinical practice, especially in the wake of COVID-19. |
| 9. | Apiratwarakul, K., et al.  Thailand | 68 | To accurately predict future incidents | Challenges:  Accurate estimation of casualty numbers is crucial in MCIs. Underestimation can lead to inadequate emergency room preparation, causing potentially avoidable casualties. Overestimation may lead to wasted resources.  There can be difficulties in manually counting the number of patients, particularly in instances where the number of casualties is high or patients are scattered.  Opportunities:  Assist in more accurately assessing MCIs by transmitting on-site information to control centres for better resource management decisions.  Effective in triage during MCIs, assisting emergency personnel in grouping patients according to the severity of symptoms.  Facilitate information transmission, allowing healthcare providers to send patient symptoms, vital signs, and initial symptom evaluations back to control centres for oversight by experienced medical directors. |
| 10. | Bin, K. J., et al. (2022).  UK | 38,042 | Evaluate the impact of a digital solution on urgent care, focusing on its ability to reduce non-essential activities, shorten nurse screening time, and decrease patient wait times for medical attention | Opportunities:  Assist in emergency care services and reduce patient waiting times.  Automate the health screening process, potentially reducing time and human error.  Reduction of non-value-added activities, streamlining healthcare services. |
| 11. | Blaivas, Blaivas and Tsung (2022) | 42 | Assessed performance impact of new ultrasound hardware on a validated DL algorithm to inferior vena case collapse | Opportunity:  Understanding and addressing the domain transfer issue can lead to more robust AI solutions that can be applied across different ultrasound machines. |
| 12. | Chen, K. W., et al. (2022).  Taiwan, China | 362 | Implement 24/7 AI-assisted STEMI detection for timely therapy | Challenge:  Existing machine-learning models for prehospital diagnosis of acute coronary syndrome (ACS) have low predictive rates or require judgment from trained EMT personnel, limiting their clinical utility.  Opportunities:  Provide cardiologist-level diagnosis, reducing the need for expert manpower in the field.  Mitigating delays due to physician-dependent reading of prehospital ECGs.  Improve chest pain triage, reduce door-to-balloon (D2B) time, and increase the percentage of D2B time under 90 minutes in emergency settings. |
| 13. | Choi, A., et al. (2022).  Korea | 468 | To accurately predict vital signs | Challenges:  Difficult to allocate patients to key treatment areas or provide standard monitoring equipment to all patients.  Standard monitoring equipment has limited mobility.  Challenging to continuously collect vital sign data manually and use it in real-time to detect deterioration in patients' clinical conditions.  Opportunities:  Accurately predict clinical deterioration and reduce time to predict septic shock.  Fill monitoring gaps potentially caused by healthcare workers' bedside visit limitations.  Reduce safety risks due to limited emergency resources. |
| 14. | Choi, J., et al. (2022).  Korea | 30 | Artificial intelligence's glottis guidance system helps novices with tracheal intubation | Opportunities:  Provide safer, faster, and more effective intubation, particularly for novices.  Certain video laryngoscopes provide better laryngeal views and improve intubation success rates.  AI-enhanced video laryngoscopes like A-LRYNGO, may further improve intubation success rates if their performance is enhanced. |
| 15. | Duanmu, H., et al. (2022). | 5766 | To accurately predict mortality and length of time on invasive mechanical ventilation | Opportunities:  ML has potential in managing resource allocation during mechanical ventilator shortages, by improving understanding of disease progression on pCXR.  Longitudinal pCXR ML analysis might predict the duration of Invasive Mechanical Ventilation (IMV) needs or mortality rates associated with COVID-19.  Deep learning of continuous imaging and non-imaging clinical data could better inform management of patients in time-sensitive, high-pressure, and potentially resource-limited environments. |
| 16. | Emakhu, J., et al. (2022).  US | 362,138 | To accurately predict acute coronary syndrome | Challenges:  Incorrect diagnosis of ACS is associated with significant morbidity and mortality, leading to unnecessary tests and increased healthcare costs.  Opportunities:  Lower mortality rates, and recognising non-cardiac causes of symptoms can reduce costs and resource usage.  Assist doctors in making decisions about additional treatments and longer stays.  The proposed framework could offer an opportunity for cost-effective diagnosis, as doctors can accurately identify patients with NSTEMI, UA, and non-ACS causes based on a broader range of symptoms. |
| 17. | Hardalaç, F., et al. (2022).  Turkey | 275 | To accurately identify and treating wrist fracture | Opportunities:  Use of AI to assist physicians in diagnosing fractures, especially in emergency services.  Help doctors via portable devices like smartphones and tablets. |
| 18. | Jha, N., et al. (2022).  Nepal, Canada | 216 | Explored medical students and interns knowledge of AI, perceptions of the role of AI in medicine, and preferences around the teaching of AI competencies | Challenges:  AI technology is still evolving and fully anthropomorphic functions are yet to be realised.  There is a lack of understanding among physicians about AI and machine learning, which could result in suboptimal patient care.  Opportunities:  AI can improve decision-making and efficiency in healthcare.  It has already shown significant utility in numerous health specialties and disease conditions, including radiology, neurosurgery imaging, skin lesions, tumors, chest pain, Alzheimer's disease, and breast cancer diagnosis.  AI can provide personalised medicine and health services.  AI can help provide preventive health advice and assist in the management of various diseases. |
| 19. | Lin, X., et al. (2022).  China | 4142(internal test)+2811(external test) | To improve rapid triage of patients presenting with chest pain | Challenges:  Lack of trained doctors for accurate diagnosis via echocardiography.  Variability in diagnosis among different expertise levels.  AI models may not be suitable for widespread use due to strict pre-image quality standards.  Maintaining image quality from portable devices.  Opportunities:  AI's powerful data processing can interpret complex medical images.  AI can perform labor-intensive tasks rapidly, saving human resources.  New models can analyse images from both standard and portable devices.  Enhances efficiency and repeatability in diagnosing myocardial infarction.  Automate image quality assessment and accurately interpret echocardiograms. |
| 20. | Rajagopalan, B., et al. (2022) |  | Improving resuscitation in cardiac arrest | Opportunity:  Enable early detection and quick activation of emergency medical services (EMS), locate nearby PADs, and guide bystanders in performing CPR and using AEDs. |
| 21. | (Yao *et al.*, 2022)  US | 4694 | Identify advanced heart failure patients for timely treatment optimisation. | Challenges:  There is a risk of exposing patients to potentially unnecessary adverse outcomes through premature or delayed delivery of therapies.  Clinicians currently rely on heuristics and evidence-based practices to determine therapy timings, which can be error-prone and ineffective for individual patients.  Most patients are managed by primary care clinicians or general cardiologists who may lack specialized training in heart transplantation and mechanical circulatory support, leading to under-recognition of disease severity and delayed referral for advanced therapies.  Opportunities:  Identify patients in need of and potentially eligible for advanced heart failure therapies, thus enabling timely referral and potentially improving patient outcomes.  Learn new clinical rules, provide transparent and accessible information, and utilise clinical knowledge to enhance its accuracy and performance.  Capture inherent uncertainties in clinical decision-making and doesn't require specific cut-off values, making it adaptable to clinical practice.  Extract new clinical knowledge and apply it to practice, thereby enhancing the continuous learning process in clinical settings.  Clinicians can examine the rules and identify any potential errors in the recommendations, allowing for real-time model updates and continuous performance improvement. |
| 22. | Zhou, L., et al. (2022).  China | 841 | To evaluate the clinical performance of an AI based motion correction reconstruction algorithm for cerebral CT | Challenges:  Uncontrolled patient head movements affect CT image quality.  Existing motion suppression methods increase workload or pose risks to patients.  Immediate rescanning for significant motion artifacts increases radiation doses and examination time.  Opportunities:  Aid in medical imaging tasks such as disease detection and image reconstruction.  Have potential to correct motion artifacts without rescanning or additional systems.  Can potentially improve overall image quality and diagnostic confidence, but their effectiveness needs further validation. |
| 23. | Arsenescu, T., et al. (2023). | 3 | Help with evaluation | Challenges:  Complexity in diagnosing arteriosclerosis requires advanced AI technologies.  Opportunities:  Improve diagnostic accuracy for arteriosclerosis.  Reduces operator dependency for image analysis.  AI combined with 3D ultrasound offers non-invasive, lower-cost diagnosis.  Early identification of plaques with AI can aid timely, personalised treatment. |
| 24. | Jeong, M., et al. (2023).  Korea | 60 | To accurately detect patient shock signs | Challenges:  AI models need a diverse array of data to predict and detect different types of shock accurately, including hemorrhagic shock, cariogenic shock, neurogenic shock, and septic shock.  Opportunities:  Assist in the early detection of shock, potentially improving patient outcomes by allowing for quicker intervention.  Predictive models based on AI could be used to identify precursors to shock, enabling preventative measures.  The use of AI could lead to more efficient patient monitoring, allowing healthcare professionals to prioritise their focus on the most critical cases. |

Supplementary Table 3: Key Findings from First Systematic Literature Review Round

| Number | Author & Year | Wearable only  Yes/No | Findings about sensor implement in A&E design |
| --- | --- | --- | --- |
| 25. | McNamara, R., et al. (2022) | No | A series of monitoring instruments and software, including the ICM+ neurological monitoring software, are used to collect and analyse high-resolution patient monitoring data in real time. These monitoring devices can capture a variety of physiological parameters, including ECG signals, Arterial Blood Pressure, Intracranial Pressure, Cerebral Perfusion Pressure, Central Venous Pressure, Pulse Oximetry, body Temperature, and brain tissue oxygen content. All of these monitoring devices should be set up beside the patient's bed, so that the patient's physiological parameters can be monitored in real time. In this setup, healthcare professionals can intervene in a timely manner based on changes in the patient's physiological status, thereby improving the therapeutic outcome for the patient. |
| 26. | Gould, J. R., et al. (2020) | No | AccuVent™ by ZOLL Medical is a state-of-the-art technology designed to provide real-time feedback on ventilation quality to healthcare professionals during resuscitation. This system comprises a differential pressure-based flow sensor positioned between the ventilation bag and the patient's airway, enabling it to measure the respiratory rate and volume of ventilation throughout the resuscitation process. The monitor is placed next to the patient for usage. The information is displayed numerically and graphically on a defibrillator or monitor. |
| 27. | Koyama, Y., et al. (2022) | yes | The capacitive pressure sensor is utilised to precisely measure the position and pressure exerted during chest compressions (CCs) in cardiopulmonary resuscitation (CPR).  In practice, the sensor is affixed to the individual. The rationale behind this positioning method is to enhance the accuracy and efficacy of chest compressions during CPR, potentially leading to improved patient outcomes. |
| 28. | Lakomek, F., et al. (2020) | yes | This study is about a real-time feedback system integrated in the corpuls3® defibrillator. The feedback sensor needs to be placed on the patient's sternum. |
| 29. | Arney, D., et al. (2023) | yes | Adding situational awareness to the clinical system by monitoring the Heart Rate Variability of clinical team members. This system uses ECG data obtained from OpenICE portable monitors carried by the clinical team members. The system processes electrocardiogram waveforms from multiple clinicians to estimate their individual cognitive load and provides real-time feedback and alerts based on changes in indicators. This process consists of six main stages: electrocardiogram data collection, electrocardiogram filtering to eliminate noise, heartbeat detection and interbeat interval calculation, HRV indicator calculation, cognitive load estimation, and alert management. |
| 30. | Garssen, S. H., et al. (2023). | yes | By using the sensor "Healthdot", vital signs are continuously measured, including Heart Rate (HR), Respiration Rate (RR), posture, and activity level to detect vibrations and displacements along different axes produced by heartbeats. As such, this sensor can monitor patient conditions in real time without reducing patient mobility and potentially lessen the workload of caregivers. The Healthdot is usually attached to the left lower rib on the midline of the patient's clavicle. The goal is to improve patient care in Acute Admission Wards (AAW) by providing early and continuous assessments, reduce the risk of patient deterioration, thus enhancing hospital efficiency. |
| 31. | Kennedy-Metz, L. R., et al. (2020) | No | This paper studies the sensors for the cognitive workload of doctors, including Heart Rate sensors and Near-Infrared Spectroscopy sensors. The heart rate sensor is a wireless device named Polar H10. Polar H10 captures and transmits all heart rate data wirelessly. Surgeons wear it on an adjustable elastic chest strap and connect it to a Bluetooth receiver (Polar V800). The NIRS sensor used is Medtronic's INVOS™ 5100C cerebral/somatic oximeter. It is applied to the forehead of the surgeons, collecting estimated values of cerebral oxygen saturation (rSO2) in the left and right prefrontal cortex (PFC) regions. The use of these sensors (especially the NIRS sensors) requires a wired connection, and it is necessary to ensure that the preamplifier is placed near the monitored surgeons and adjust its position as the surgeons move around the operating table. The INVOS™ monitor itself must also be placed a short distance from the preamplifier and kept on the heart-lung bypass pump. |
| 32. | Tomita, K., et al. (2019) | No | The study is about a non-invasive monitoring device used in ICU and operating rooms. This device uses a single sensor to continuously measure blood pressure, pulse rate, respiration rate, and oxygen saturation. The sensor operates using photoplethysmography technology. The device has been developed as a compact, wireless, single-sensor system capable of continuously monitoring these four physiological parameters. The monitoring device includes a transmitter, a sensor, and a charging cable. It requires a display to be used in conjunction, connected to the transmitter via Bluetooth. |
| 33. | Polley, C., et al. (2021) | yes | This research proposes an innovative wearable triage system, aiming to enhance the efficiency and effectiveness of patient assessment processes within Emergency Departments and reduce the workload of triage nurses. The device is designed as an integrated sensor system capable of wirelessly detecting respiration and cardiac activity in patients. |
| 34. | Choi, A., et al. (2022) | Yes | Primarily used in Emergency Departments for the treatment of patients with fever but in stable conditions. By continuously monitoring vital signs, the device aims to detect and predict clinical deterioration in patients at risk of septic shock earlier. The sensor used in this study is Hicardi, which is used to continuously monitor and record single-lead ECG, respiration rate and skin surface temperature, as well as patient position and activity. These vital signs are continuously monitored by a device installed on the patient's chest, enabling up to 24 hours of uninterrupted data collection. Then, the sensor processes these signals and transmits them to a mobile device equipped with the corresponding smartphone application via Bluetooth. |
| 35. | Satake, S., et al. (2019) | No | A non-invasive continuous arterial pressure monitoring device that uses microelectromechanical system (MEMS). The device is developed to provide a safer, more convenient alternative to invasive blood pressure monitoring methods. The sensing diaphragm of this device is pressed directly on the radial artery above the skin. The pulse of the artery causes a distortion in the shape of the diaphragm, which is then converted into voltage by the MEMS. The voltage measured by the MEMS sensor is amplified through a signal amplification circuit and transmitted to the monitor display. This device can monitor arterial pressure continuously and non-invasively, having potential applications in critical care situations such as Emergency Departments and Intensive Care Units where continuous blood pressure monitoring is necessary. |
| 36. | Wang, H. W., et al. (2022) | No | This paper investigates the use of CCTV cameras for continuous cardiopulmonary monitoring of critically ill patients in the ICU. CCTV surveillance cameras can remotely optically measure subtle skin colour changes caused by arterial pulsation using a camera-based photoplethysmography method. This technology allows for non-contact heart rate measurement. Similarly, the camera can measure the motion of the intercostal muscles of the chest and abdomen caused by inhalation and exhalation and calculate it as the breathing rate. The camera is installed on the ceiling facing the patient, ensuring the upper half of the patient is in the centre of the video frame.  The application of the camera does not require any contact with the patient, thereby overcoming some drawbacks of traditional contact patient monitoring devices. The non-contact nature of this method reduces interference with the care workflow in tasks such as cleaning the patient's body or changing dressings. It also provides the potential to simultaneously monitor multiple patients. |

Supplementary Table 4: Key Findings from Second Systematic Literature Review Round
